# Exploring the cross-sectional association between the strength of school vaping policies and student vaping behaviours using data from the 2021–2022 COMPASS Study

**DOI:** 10.17269/s41997-024-00919-0

**Published:** 2024-07-29

**Authors:** Erin Kostuch, Richard Bélanger, Scott T. Leatherdale, Adam G. Cole

**Affiliations:** 1 Faculty of Health Sciences, Ontario Tech University, Oshawa, ON Canada; 2 Faculté de Médecine, Université Laval, Québec, Québec Canada; 3 School of Public Health Sciences, University of Waterloo, Waterloo, ON Canada

**Keywords:** Youth/adolescent, Vaping; E-cigarette use, School policy, Jeunes/adolescents, vapotage, usage de cigarettes électroniques, politique scolaire

## Abstract

**Objectives:**

Youth vaping is a concern in Canada. While school-level policies influence student behaviours, few studies have investigated the association between school vaping policies and student vaping. This study reviewed and scored the comprehensiveness of school vaping policies and investigated the association between school vaping policy scores and student vaping.

**Methods:**

Online policy documents from *n* = 39 schools in Ontario, Alberta, and British Columbia, Canada, participating in the 2021–2022 wave of the COMPASS study were collected, reviewed, and scored for comprehensiveness (/39) using the School Tobacco Policy Index (STPI) rating form. The mean and range of scores for each domain of the STPI were calculated. School policy scores were linked to student vaping data from the COMPASS study. Multilevel logistic regression analyses identified the association between school vaping policy score and student lifetime and current (past 30-day) vaping.

**Results:**

The mean total policy score was 10.2/39 (range 0‒24), and 28% of schools scored 0/39. The majority of school policies did not identify enforcement approaches or available preventive or cessation resources. Increasing STPI score was not associated with the odds of student lifetime or current vaping in multilevel logistic regression analyses.

**Conclusion:**

The STPI quickly identified components of school vaping policies that were missing. The overall score of most school vaping policies in our sample was low and most school vaping policies lacked many important components. Future studies should explore factors associated with adolescent vaping and identify effective prevention measures.

**Supplementary Information:**

The online version contains supplementary material available at 10.17269/s41997-024-00919-0.

## Résumé

**Objectifs:**

Le vapotage des jeunes est préoccupant au Canada. Les politiques scolaires influencent les comportements des élèves, mais il y a peu d’études sur l’association entre les politiques scolaires sur le vapotage et le vapotage des élèves. Nous avons passé en revue des politiques scolaires sur le vapotage et noté leur complétude, puis étudié l’association entre les notes attribuées aux politiques scolaires sur le vapotage et le vapotage des élèves.

**Méthode:**

Les documents de politique accessibles en ligne de *n *= 39 écoles de l’Ontario, de l’Alberta et de la Colombie-Britannique, au Canada, ayant participé au cycle 2021-2022 de l’étude COMPASS ont été collectés, passés en revue et notés selon leur complétude (/39) à l’aide du formulaire de notation de l’indice School Tobacco Policy Index (STPI). La moyenne et l’intervalle des notes attribuées pour chaque élément de l’indice STPI ont été calculés. Les notes des politiques scolaires ont été maillées aux données sur le vapotage des élèves de l’étude COMPASS. Des analyses de régression logistique multiniveaux ont repéré l’association entre les notes attribuées aux politiques scolaires sur le vapotage et le vapotage des élèves à un moment de leur vie et récemment (au cours des 30 jours précédents).

**Résultats:**

La note totale moyenne attribuée aux politiques a été de 10,2/39 (intervalle : 0‒24), et 28 % des écoles ont obtenu une note de 0/39. La majorité des politiques scolaires ne mentionnaient pas d’approches ou de ressources de prévention et d’arrêt disponibles. Les notes STPI plus élevées n’étaient pas associées à la probabilité de vapotage récemment ou à un moment de la vie des élèves dans les analyses de régression logistique multiniveaux.

**Conclusion:**

L’indice STPI a rapidement repéré les lacunes dans les politiques scolaires sur le vapotage. La note globale de la plupart des politiques scolaires sur le vapotage de l’échantillon était faible, et il manquait d’importants éléments dans la plupart des politiques. Les études futures devraient explorer les facteurs associés au vapotage des adolescents et identifier des mesures de prévention efficaces.

## Introduction

The prevalence of vaping among adolescents in Canada has rapidly increased since the introduction of e-cigarettes and other vaping devices to the Canadian market in 2004 (CAMH, 2019; Cole et al., 2020; Reid et al., 2022). Nationally representative data indicate that the prevalence of past 30-day vaping among adolescents 15–19 years old increased from 2.6% in 2013 to 14.4% in 2020 (Reid et al., 2022). According to the 2022 Canadian Tobacco and Nicotine Survey, 30.0% of adolescents aged 15–19 years old have ever tried vaping, 13.6% vaped in the last 30 days, and 6.5% vaped daily (Government of Canada & Statistics Canada, 2023). Despite being marketed as safer than cigarettes, the aerosol from vapes can harm the respiratory and cardiovascular systems (Marques et al., 2021; Skotsimara et al., 2019; Wills et al., 2021) and nicotine is highly addictive (U.S. Department of Health & Human Services, 2014).

Since substance use initiation typically begins in adolescence, school environments have the potential to greatly influence student vaping behaviours (Leatherdale et al., 2014). Most adolescents spend approximately 25 h per week in school where vaping may be displayed as normative behaviour by other students, staff, and/or visitors (Cho et al., 2019; Milicic et al., 2018). In fact, evidence from the United States suggests that in 2019, two thirds of students had ever seen anyone vaping in or around the school, and seeing someone vaping at school was associated with a higher likelihood of lifetime vaping and susceptibility to future vaping (Dai, 2021; Mantey et al., 2021). An environment where the use of vapes is not permitted may help to prevent adolescent vaping; this makes schools an ideal place to enact and enforce anti-vaping policies (Cole et al., 2019; Galanti et al., 2014; Patel et al., 2022).

Vaping policies and their presentation to students and school staff can vary across schools, which can impact student and staff knowledge and awareness of school vaping policies. A 2020 Health Canada report revealed that many school staff were not well informed about school-level vaping policies, leading to inconsistent approaches to control vaping in schools (Health Canada, 2020). Other evidence from the USA also indicates variability in school staff knowledge and awareness of vaping devices and school policies regarding vaping (Schillo et al., 2020). Only one study has examined the influence of school vaping policies on student behaviours and it found that a provincial policy banning vaping on school property reduced the likelihood of student vaping (Milicic et al., 2018). Since school policies can contribute to the community acceptance of vaping by influencing social and environmental norms within the school, it is important to review the comprehensiveness of school vaping policies (Boyce et al., 2009; Leatherdale et al., 2014).

The School Tobacco Policy Index (STPI) rating form is a standardized tool to evaluate the strength and comprehensiveness of school tobacco policies, focusing on traditional tobacco products (e.g. cigarettes) (Boyce et al., 2009). While a previous study investigated the association between school tobacco policy scores and youth cigarette smoking (Cole et al., 2019), the STPI has not been applied to evaluate the strength of school vaping policies. Exploring how the STPI can be extended to assess school vaping policies can support schools in creating strong policies against vaping and could prevent youth vaping. The purpose of this study was to review and score the comprehensiveness of school vaping policies using the STPI and examine the cross-sectional association between school vaping policy score and lifetime and current vaping.

## Methods

### Vape policy search

Consistent with a previous evaluation of school tobacco policies (Cole et al., 2019), an online search for vaping policy documents for *n* = 69 English-language schools from British Columbia (*n* = 13), Alberta (*n* = 5), and Ontario (*n* = 51) participating in the COMPASS study in 2021–2022 was conducted between May 6 and 13, 2022, using the Google search engine. Given the increased frequency with which schools share information with their school community online, we expected to find many policy documents through a web search.

The COMPASS study is a prospective cohort study that collects student health behaviour data from a convenience sample of Canadian high school students in grades 9–12 and the schools they attend in order to evaluate how changes in school programs, policies, and environments impact youth health behaviours and outcomes over time (Leatherdale et al., 2014). Additional information about the sampling and recruitment methods is available online (https://uwaterloo.ca/compass-system/). Briefly, all English-language public and private school boards with schools with grades 9–12, with a student population of at least 90 students per grade, that operated in a standard school/classroom setting, and that permitted the use of active-information passive-consent procedures were invited to participate in the COMPASS study (Thompson-Haile et al., 2013). In this consent procedure, the parents/guardians of all students in the school were provided information about the study and contacted the research team if they did not want their student to participate (Thompson-Haile et al., 2013). Targeting school boards and schools that allowed this consent procedure maximized student participation and limited response bias associated with active consent studies (Thompson-Haile et al., 2013). All students in grades 9–12 in recruited schools were eligible to participate, and students could choose whether or not to complete the survey the day of the data collection. The COMPASS study sample is not designed to be provincially or nationally representative; however, tobacco use and vaping rates are consistent with provincial estimates. The COMPASS study received ethics approval from the University of Waterloo Research Ethics Board (ORE #30118) and participating school boards, and this secondary data analysis received ethics approval from the Ontario Tech Research Ethics Board (#17240).

We used the search terms “student handbook” and “student code of conduct” to identify relevant documents for each participating school. The identity of each school was verified by confirming their address on their website and/or policy documents. The status of each policy was confirmed by locating the school year (2021–2022) within the handbooks and policy documents; however, school documents without a specified date (*n* = 14) were also included. School vaping policies were identified within each document by searching the key words “vaping”, “vape”, and “e-cigarette” throughout all sections. This was to ensure all information pertaining to a vaping policy was identified as different policy components could be found in multiple sections.

### Instruments

#### School Tobacco Policy Index (STPI)

The STPI is a tool that was designed to provide a standardized approach to evaluate the strength and comprehensiveness of school and school board tobacco policies by assessing whether or not “gold standard” components are included (tool available online: https://openscholarship.wustl.edu/cphss/43/). Strong and comprehensive school and school board tobacco policies will explicitly include more components listed in the tool. The STPI evaluates the strength of school tobacco policies across four domains (Boyce et al., 2009; Center for Tobacco Policy Research & Moreland-Russell, 2005). To apply this tool to assess the strength of school vaping policies, we changed components of the STPI to assess “vaping” rather than “tobacco”. We removed one item from the STPI (*Domain 4: Policy Organization*: Policy indicates all tobacco products) because it was no longer relevant given the focus on vaping policy. A brief description of each domain in the tool is provided below.

##### Domain 1

Vape-Free Environment (14 possible points): Points are awarded for policies that state that school buildings, grounds, and events are vape-free for students, staff, and visitors.

##### Domain 2

Enforcement (12 possible points): Points are awarded for policies that describe enforcement activities and consequences for students, staff, and visitors who violate the policy.

##### Domain 3

Prevention and Treatment Services (6 possible points): Points are awarded for policies that describe vaping prevention curricula for students and vape cessation services for students and staff.

##### Domain 4

Policy Organization (7 possible points): Points are awarded for policies that explain how the policy is communicated, the rationale for the policy, and the management of the policy.

Each policy was reviewed according to the STPI instructions, and one point was awarded for each component within each domain that was explicitly stated within the school policy, to a maximum score of 39.

#### COMPASS student questionnaire

Cross-sectional student-level data from the 2021–2022 school year (Year 10 of the COMPASS study) were collected through the COMPASS questionnaire (Cq), an online survey that was completed by students. The vast majority (95%) of students completed the survey during class time; the remaining students completed the survey outside of class time (Rezvani et al., 2023). Average student participation rates across schools were generally high (> 70%). Consistent with other surveys of youth health behaviours, lifetime vaping was measured with the question “Have you ever tried a vape, also known as an e-cigarette? (e.g., JUUL, Vype, Suorin, Smok)”. Students who answered “yes” were categorized as ever users, while those who answered “no” were categorized as never users. Current vaping was measured with the question “On how many of the last 30 days did you use a vape?”. Students who reported vaping on one or more days within the last 30 days were categorized as current users, while those who did not vape in the last 30 days were non-current users. The Cq also collected relevant sociodemographic information, including student gender (boy/man, girl/woman, other, I prefer not to say/not stated), grade (9, 10, 11, 12), ethnicity (White, Black, East Asian, Latino, Middle Eastern, South Asian, Southeast Asian, other, multiethnic, and prefer not to say/not stated), and amount of weekly spending money ($0, $1–$20, $21–$100, > $100, I don’t know).

### Sample selection

We identified policy documents from 56.5% of schools that participated in 2021–2022 (39/69 schools), including schools from British Columbia (*n* = 2), Alberta (*n* = 3), and Ontario (*n* = 34). The mean school-level prevalence of lifetime vaping was not significantly different between schools with (34.7%) or without a policy score (35.8%, *t* = 0.43, *p* = 0.668). Similarly, the mean school-level prevalence of current vaping was not significantly different between schools with (19.8%) or without a policy score (20.6%, *t* = 0.39, *p* = 0.700). The sample included *n* = 27,003 students from these provinces; we excluded students with missing vaping behaviour data (*n* = 3021) or grade (*n* = 46) and students at schools where we could not find vaping policy documents (*n* = 9657). We also excluded *n* = 1359 students who attended online schools as a result of the COVID-19 pandemic, since a school vaping policy would not apply to these students. This resulted in an analytic sample of *n* = 12,920 students from *n* = 39 schools with policy scores. Demographic characteristics of the student sample can be found in Online Resource 1.

### Analysis

Vaping policies were reviewed using the STPI and then scored for a total out of 39. First, two individuals independently scored three policies; differences were discussed to clarify the interpretation and application of the STPI, and then a single reviewer scored the remaining policy documents. The mean and range of scores across the sample of schools for each domain of the STPI were calculated.

Two multilevel logistic regression models investigated the association between STPI score (a school-level factor) and student lifetime (model 1) and current (past 30-day) vaping (model 2). Multilevel logistic regression models account for the clustered nature of the data (students clustered within schools), and provide more precise parameter estimates, standard errors, confidence intervals, and significance tests (Guo & Zhao, 2000). A 4-unit increase in the STPI score was modelled as this would represent an increase in the score in each domain. Both models controlled for student-level clustering within schools, the province of the school, and student sociodemographic characteristics known to be associated with student vaping and substance use (i.e. gender, grade, ethnicity, and amount of weekly spending money). The data analysis for this study was generated using SAS software, Version 9.4 of the SAS System for Windows.

## Results

### School vaping policy scores

Overall, school vaping policy scores in our sample were low; 77% of schools (*n* = 30) scored below 20/39 and 28% (*n* = 11) of schools scored 0/39. The mean total policy score was 10.2/39 , with a range of 0 (*n* = 11 schools) to 24 (*n* = 4 schools). All four domains had both a minimum and a mode score of 0 (Table 1). The sections that follow summarize key components of each domain of the STPI that were present and absent from school vaping policies.

#### Domain 1: Vape-Free Environment

Most school policies clearly prohibited student vaping in school buildings (72%) and outside on school grounds (67%). Only 28% of policies explicitly restricted vaping in school buildings or outside on school grounds for staff and visitors. Few policies (13%) stated that vaping was prohibited at all times (24 h a day and/or 365 days a year).

#### Domain 2: Enforcement

Most school policies mentioned that the policy would be enforced for students (69%) and mentioned specific consequences for violations (67%). Only 26% of policies mentioned that the policy would be enforced for staff and/or visitors, and 21% mentioned specific consequences for staff/visitors who violated the policy. The types of consequences mentioned included warnings, suspension, fines from local health units, detention, confiscation, service work, loss of privileges, and cessation services within the school as well as referrals to outside services. The most common approaches among schools were suspension and fines from local health units. In schools that listed suspensions and fines, none of the policies described the length of the possible suspension, and only *n* = 11 provided a specific amount for the fine based on provincial regulations ($200–$5000). About half of policies (49%) mentioned a specific individual who would be responsible for enforcing the policy.

#### Domain 3: Prevention and Treatment Services

None of the school policies mentioned specific school prevention curricula addressing vaping. Only one policy discussed cessation support and referral to outside treatment services for students.

#### Domain 4: Policy Organization

Only 21% of policies provided a rationale for the development of the policy, such as health or environmental consequences of vaping. None of the policies referenced signs prohibiting vaping within the school, and none of the policies referenced an individual to review or update the policy.

#### Examples of vaping policies

**Fig. 1:**
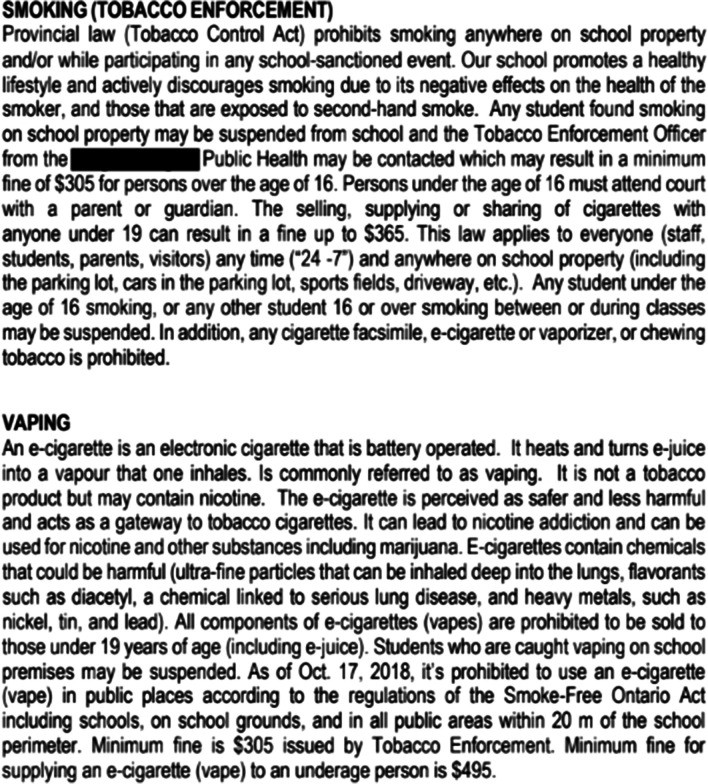
Example of a high-scoring school vaping policy

**Fig. 2:**
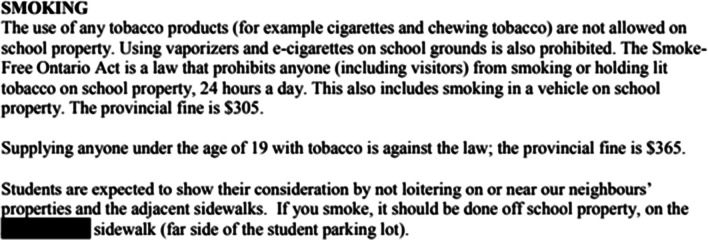
Example of a low-scoring school vaping policy

The highest scoring vaping policy (24/39) was found in four school handbooks within the same Ontario school board. As shown in Fig. 1, this policy included a general overview of vaping, health consequences of vaping, and possible disciplinary consequences from both the school and the local health unit for students caught vaping. In contrast, one of the lowest scoring vaping policies (5/39) only mentioned that vaping is prohibited on school property and that there are provincial laws against vaping on school property, but did not indicate specific school consequences for students caught vaping (Fig. 2).

### Association between school vaping policy score and student vaping

In 2021–2022, approximately one third (33.7%) of students in our sample reported lifetime vaping, while 18.8% reported current (past 30-day) vaping. As shown in Table 2, there was no association between increasing STPI score and student lifetime or current vaping.

## Discussion

The School Tobacco Policy Index (STPI) is a simple tool that can quickly identify missing areas within school tobacco policies. By applying the STPI to evaluate vaping policies in schools, this study identified aspects that could be improved in a sample of Canadian schools and whether increasing policy score was associated with a reduced likelihood of student vaping. The mean total policy score 10.2/39 and the low range of STPI scores (0–24) suggest that most school vaping policies lack many important components; 28% of schools in our sample did not include any vaping-relevant information in their policy documents. We did not identify an association between increasing vaping policy score and the likelihood of lifetime or current vaping among students.

Provincial laws prohibit vaping in and around schools (Tobacco, Smoking and Vaping Reduction Act, 2021; Tobacco and Vapour Products Control Act, 2023; Smoke-Free Ontario Act, 2017), but schools with no vaping policy convey an inconsistent message regarding these laws. Because the STPI measures components of a “gold-standard” policy recommendation, it is important that schools develop and implement vaping policies that meet and even exceed the requirements of provincial laws in order to restrict vape use and exposure among students (Boyce et al., 2009; Canadian Paediatric Society, 2021). STPI scores lower than 20 indicate that a policy does not contain as many important vaping control elements as possible (Boyce et al., 2009). In this study, *Domain 1: Vape-Free Environment* and *Domain 4: Policy Organization* scored the highest, which may indicate a general focus on school environments and broad communication of policies through written materials such as student handbooks and school websites. However, the rationale for vaping policies including health and/or environmental consequences (a component of *Domain 4: Policy Organization*) was only provided in 21% of school policies. Such information is important for communicating why school vaping policies exist to the school community. In addition, *Domain 2: Enforcement* and *Domain 3: Prevention and Treatment Services* scored the lowest. This suggests that even the most comprehensive policies in our sample may not be effective in discouraging student vaping. Only one school policy discussed cessation support and referral to outside treatment services, which may indicate a lack of school- and community-based vaping cessation support services for students and school staff.

Many of the school policies examined in our sample did not state specific consequences for vaping policy violations, but instead discussed mitigating factors such as the student’s age, level of maturity, and level of risk to other students when considering what consequence to impose. While many of these factors should be considered by school administrators when enforcing school vaping policies, previous evidence from the USA indicates that many school staff are not sure of the school’s vaping policy (Schillo et al., 2020) and do not see any consequences for students found vaping at school (Cole et al., 2022). A lack of clear consequences for students found vaping at school may impede enforcement efforts and contribute to higher vaping rates at a school. Clearly outlining specific disciplinary actions for those who violate the school’s policies, consistently enforcing school substance use policies, and incorporating education and cessation resources into both preventive and disciplinary actions may improve policy effectiveness and reduce student vaping at school (Collier et al., 2019; Galanti et al., 2014).

The regression analyses did not identify an association between school vaping policy score and student vaping. Previous research indicates that comprehensive school policies influence student health behaviours (Cole et al., 2019; Galanti et al., 2014; Leatherdale et al., 2014; Patel et al., 2022). The low school vaping policy scores identified in this study suggest that many school vaping policies may not be comprehensive, lessening the potential positive impact on student vaping. Future research should continue to examine the strength of school vaping policies and changes to school vaping policies and their influence on student vaping behaviours. To better support schools, public health organizations and professionals could use the STPI to identify policy components that school vaping policies are lacking and provide guidance to strengthen the school’s vaping policies. An example of a “gold standard” school vaping policy that includes all of the components within each domain of the STPI is provided in the Appendix.

### Strengths and limitations

To our knowledge, this is the first study to examine and score the strength of school vaping policies using an established and structured tool. The STPI provides clear criteria for evaluating and scoring school policies and recommendations for how to improve them. A strength of the current study was the ability to link school vaping policy scores and student behaviours. The use of active-information passive-consent procedures in the COMPASS study limits selection bias (i.e. students engaged in risky behaviours are less likely to return permission forms in active consent studies) and provides more robust school-level data (Liu et al., 2017; Thompson-Haile et al., 2013).

The largest limitation to this study was the small convenience sample of school vaping policies evaluated. Despite our online search, we were unable to identify school policies for all included COMPASS schools. As this was a secondary data analysis, we were not able to follow up with schools where we could not identify policy documents. Such follow-up would have taken place after the student data were collected and may not accurately represent the vaping policy at the school at the time of the data collection. Some schools may have vaping policies but not post the information online. A sample size of 39 high schools may not provide a comprehensive picture of school vaping policies within each province or across Canada. Additionally, the schools selected within the COMPASS study represent a convenience sample and therefore do not represent all schools in British Columbia, Alberta, Ontario, or Canada. Additional data from other provinces and territories are necessary to evaluate whether these results are consistent across Canadian high schools. This was a cross-sectional analysis; longitudinal data are needed to evaluate how changes in school vaping policies impact student behaviours.

## Conclusion

We applied the STPI to quickly identify components of school vaping policies that were missing. The overall score of most school vaping policies in our sample was low and most policies lacked specific components that are vital to the reduction and prevention of student vaping, including clear consequences for students found vaping at school and vaping prevention and cessation resources. While stronger school vaping policies may help reduce student vaping, additional research is needed to further explore factors associated with adolescent vaping and identify effective preventive measures.

## Contributions to knowledge

What does this study add to existing knowledge?

- There are few studies that have examined the association between school vaping policy and student vaping.
- School vaping policy scores were very low, indicating that most school vaping policies were missing important components that could discourage vaping among students.
- Few school vaping policies referenced prohibiting vaping for staff and visitors, only one discussed cessation support for students, and few provided a rationale for the policy.

What are the key implications for public health interventions, practice, or policy?

- Given that most school vaping policy scores were low and were missing important components that could discourage vaping among students and school staff, schools may need support in developing comprehensive school vaping policies and intervening with students who vape.
- The School Tobacco Policy Index is a useful tool to quickly identify missing areas within school tobacco and vaping policies.

## Electronic supplementary material

Below is the link to the electronic supplementary material.

Supplementary file1 (DOCX 9 KB)

## Data Availability

COMPASS data are available upon reasonable request by completing a COMPASS Data Usage Application at: https://uwaterloo.ca/compass-system/information-researchers

## Appendix

### Example school vaping policy

Vapes (also known as e-cigarettes) are battery-powered devices that heat a liquid that contains nicotine or other substances into an aerosol that is inhaled. Vaping can be addictive, damage the lungs, and harm those who do not vape through second-hand exposure to aerosol. To protect the health of our school community, vaping is prohibited anywhere on school property and while participating in any school-sanctioned event (e.g. field trip, school dance). This policy applies to everyone (staff, students, parents, and visitors), 24 h/day, 365 days/year, and anywhere on school property (including inside school buildings, on the school parking lot, on the sports fields, on the driveway, etc.). Students and staff are also prohibited from wearing or carrying clothing or accessories that display vape industry brand names or logos on school property.

#### Consequences for vaping at school

The school Principal/Vice-Principal is responsible for enforcing this policy. Vape devices will be confiscated from any student or staff who vapes on school property. Parents/guardians may need to meet with administration before the device will be returned to a student. Students who vape on school property will receive a minimum 1-day suspension for a first offence, and a minimum 3-day suspension for subsequent offences. Students, staff, and visitors who vape on school property can also be fined a minimum $305 by a Tobacco Enforcement Officer.

#### Resources and support

Our school supports the health and well-being of students and staff. The provincial curriculum provides opportunities for students to learn about vaping to make healthier choices for their overall well-being. Students who would like to reduce or stop vaping are encouraged to speak with a teacher, school counsellor, or coach who can listen and recommend resources for support. Students can also download the Quash app (https://www.quashapp.com/) to create a customized quit plan. Staff who would like to reduce or stop vaping can contact the Employee Assistance Program for support.

This policy will be reviewed annually by the school Principal/Vice Principal. This policy was last reviewed January 2024.

**Table 1:** Summary statistics for vaping policy scores from the STPI for high schools participating in the 2021–2022 COMPASS study

School Tobacco Policy Index Domain	*N*	Mean score	Minimum score	Maximum score
1. Vape-free environment (/14)	39	5.0	0	14
2. Enforcement (/12)	39	2.8	0	7
3. Prevention and treatment services (/6)	39	0.05	0	2
4. Policy organization (/7)	39	2.3	0	4

**Table 2:** Odds ratio estimates for the association between school vaping policy score and student vaping, 2021–2022 COMPASS study

Model^a^	Adjusted odds ratio (95% CI)
Lifetime vaping	0.99 (0.94, 1.05)
Current vaping	0.98 (0.91, 1.04)

^a^Both models controlled for student-level clustering within schools, province of the school, and student gender, grade, ethnicity, and weekly spending money
