# Supplementary material for: Exploring the cross-sectional association between the strength of school vaping policies and student vaping behaviours using data from the 2021–2022 COMPASS Study
Source: Can J Public Health. 2024 Jul 29;115(6):936–45. doi: 10.17269/s41997-024-00919-0 (PMC11638446; doi:10.17269/s41997-024-00919-0)
Supplement: Supplementary file 1 — Supplementary file1 (DOCX 9 KB) [file 41997_2024_919_MOESM1_ESM.docx]

**Online Resource 1**. Demographic characteristics of the analytic sample, 2021-22 COMPASS study (n=12,902)

| **Characteristic** | **% (n)** |
| --- | --- |
| Gender |  |
| Girl/woman | 46.8 (6040) |
| Boy/man | 46.7 (6029) |
| Other | 4.5 (579) |
| I prefer not to say / Not Stated | 2.1 (272) |
| Grade |  |
| 9 | 27.6 (3569) |
| 10 | 29.7 (3843) |
| 11 | 24.1 (3117) |
| 12 | 18.5 (2391) |
| Ethnicity |  |
| White | 59.2 (7651) |
| Black | 5.1 (657) |
| East Asian | 5.0 (640) |
| Latino | 2.8 (395) |
| Middle Eastern | 2.3 (295) |
| South Asian | 3.0 (387) |
| Southeast Asian | 4.8 (625) |
| Multiethnic | 8.9 (1147) |
| Other | 4.2 (545) |
| I don’t know / I prefer not to say / Not Stated | 4.7 (608) |
| Amount of weekly spending money |  |
| $0 | 23.6 (3047) |
| $1-$20 | 17.3 (2240) |
| $21-$100 | 19.5 (2524) |
| >$100 | 22.1 (2861) |
| I don’t know / Not Stated | 17.4 (2248) |
